# The transcription factor Nurr1 is upregulated in amyotrophic lateral sclerosis patients and SOD1-G93A mice

**DOI:** 10.1242/dmm.043513

**Published:** 2020-05-15

**Authors:** Valeria Valsecchi, Marina Boido, Francesca Montarolo, Michela Guglielmotto, Simona Perga, Serena Martire, Santina Cutrupi, Andrea Iannello, Nadia Gionchiglia, Elena Signorino, Andrea Calvo, Giuseppe Fuda, Adriano Chiò, Antonio Bertolotto, Alessandro Vercelli

**Affiliations:** 1 Department of Neuroscience Rita Levi Montalcini, University of Turin, via Cherasco 15, 10126 Turin, Italy; 2 Neuroscience Institute Cavalieri Ottolenghi (NICO), University of Turin, Regione Gonzole 10, 10043 Orbassano, Turin, Italy; 3 Department of Neuroscience, Reproductive and Dentistry Sciences, University of Naples “Federico II”, via S. Pansini 5, 80131, Naples, Italy; 4 Neurobiology Unit, Neurology - CReSM (Regional Referring Center of Multiple Sclerosis), AOU San Luigi Gonzaga, Regione Gonzole 10, 10043 Orbassano, Turin, Italy; 5 Department of Clinical and Biological Sciences, University of Turin, Regione Gonzole 10, 10043 Orbassano, Turin, Italy; 6 Department of Neuroscience Rita Levi Montalcini, Amyotrophic Lateral Sclerosis Expert Center (CRESLA), University of Turin, via Cherasco 15, 10126 Turin, Italy; 7 University Hospital Città della Scienza e della Salute, corso Bramante 88, 10126 Turin, Italy

**Keywords:** ALS, SOD1-G93A mice, Motor neuron disease, Neuroinflammation, Nurr1

## Abstract

Amyotrophic lateral sclerosis (ALS) is a neurodegenerative disease that affects both lower and upper motor neurons (MNs) in the central nervous system. ALS etiology is highly multifactorial and multifarious, and an effective treatment is still lacking. Neuroinflammation is a hallmark of ALS and could be targeted to develop new therapeutic approaches. Interestingly, the transcription factor Nurr1 has been demonstrated to have an important role in the inflammatory process in several neurological disorders, such as Parkinson's disease and multiple sclerosis. In the present paper, we demonstrate for the first time that Nurr1 expression levels are upregulated in the peripheral blood of ALS patients. Moreover, we investigated Nurr1 function in the SOD1-G93A mouse model of ALS. Nurr1 was strongly upregulated in the spinal cord during the asymptomatic and early symptomatic phases of the disease, where it promoted the expression of brain-derived neurotrophic factor mRNA and the repression of NFκB pro-inflammatory targets, such as inducible nitric oxide synthase. Therefore, we hypothesize that Nurr1 is activated in an early phase of the disease as a protective endogenous anti-inflammatory mechanism, although not sufficient to reverse disease progression. On the basis of these observations, Nurr1 could represent a potential biomarker for ALS and a promising target for future therapies.

## Abstract (precis)

**Summary:** We hypothesize that the transcription factor Nurr1 is activated in the early phase of the neurodegenerative disease amylotrophic lateral sclerosis (ALS), probably as a neuroprotective endogenous mechanism. Nurr1 might represent a promising target for ALS therapy.

## INTRODUCTION

Amyotrophic lateral sclerosis (ALS) is a neurodegenerative disease that affects both lower motor neurons (MNs) in the brainstem and spinal cord and upper MNs in the cerebral cortex (Pasinelli and Brown, 2006). Loss of these neurons leads to muscle weakness and paralysis and ultimately to death owing to respiratory failure 2-5 years after diagnosis (Worms, 2001). The incidence of the disease is about 1 to 2 individuals per 100,000 per year, with males being affected more frequently than females (Robberecht and Philips, 2013). Considering all cases, 90% are classified as sporadic (sALS) and the remaining 10% as familial (fALS) with a Mendelian pattern of inheritance. In fALS, mutations have been identified in genes encoding several proteins: the copper/zinc superoxide dismutase 1 (SOD1), the transactive response DNA-binding protein (TARDBP), the fused in sarcoma/translocated in liposarcoma RNA-binding protein (FUS/TLS) and the chromosome 9 open reading frame 72 (C9orf72). Mutations in these genes account for two-thirds of all fALS cases and for approximately one-tenth of sALS cases, although for other incidences the etiology is unknown (Renton et al., 2014; Chia et al., 2018). The clinical phenotype of fALS is usually indistinguishable from sALS and an effective therapy is still elusive.

ALS etiology is multifarious and has been associated with many factors: mitochondrial dysfunction and oxidative stress, excitotoxicity, deficits in axonal architecture/function and protein quality control, protein misfolding and aggregation, loss of neurotrophic factors, dysregulation of RNA metabolism and inflammation (Ferraiuolo et al., 2011; Cozzolino et al., 2012). In particular, neuroinflammation is a common hallmark of neurodegenerative diseases, such as ALS and Parkinson's disease (PD). Indeed, in ALS a dramatic activation of microglia, astrocytes and the complement system is reported, contributing to neurodegeneration partly as a consequence of the upregulation of inflammatory genes (Haidet-Phillips et al., 2011). In addition, the role of the adaptive immune system modulates the balance between neuroprotection and neurotoxicity (Thonhoff et al., 2018). The effects on the central nervous system (CNS) and peripheral blood alter over the course of the disease and the dysregulation of regulatory T cells affects the disease outcome (Henkel et al., 2009). Hence, as neuroinflammation makes a central contribution to ALS pathogenesis, the identification of novel therapeutic targets acting on inflammation is considered mandatory (Troost et al., 1990; Zhao et al., 2013).

In this scenario, an interesting role is played by the nuclear receptor related 1 protein (Nurr1), also called NR4A2 (encoded by the gene *NR4A2*). Nurr1 is an orphan receptor belonging to the nuclear receptor subfamily 4 group A (NR4A) family, which also includes the nerve growth factor IB (NGFIB, Nur77, NR4A1) and the neuron-derived orphan receptor 1 (NOR-1, NR4A3). In the CNS, Nurr1 has a well-established role in the development and maintenance of midbrain dopaminergic (mDA) neurons (Zetterström et al., 1997; Saucedo-Cardenas et al., 1998). Given its crucial functions, altered Nurr1 expression has been implicated in dopamine-associated brain disorders, including PD. Notably, the expression of Nurr1 in mDA neurons decreases in PD patients (Hering et al., 2004) and single-nucleotide polymorphisms (SNPs) and mutations resulting in reduced expression of Nurr1 are associated with familial and sporadic forms of PD.

Interestingly, Nurr1 is a constitutively active transcription factor binding its target genes as a monomer, homodimer or heterodimer in association with retinoid X receptors (Aarnisalo et al., 2002; Maira et al., 1999; Wang et al., 2003). In murine models of PD, Nurr1 was found to have roles in both neuroprotection and immunomodulation. Indeed, Nurr1 has an anti-inflammatory role, inhibiting expression of the genes encoding pro-inflammatory components of the NFκB pathway in microglia and astrocytes (Saijo et al., 2009). Specifically, knocking down Nurr1 in mice leads to an increased activation of glial cells exposed to lipopolysaccharide (LPS), with subsequent production of higher levels of mRNAs encoding inflammatory cytokines and neurotoxic effector proteins such as tumor necrosis factor α (TNF-α), inducible nitric oxide synthase (iNOS) and interleukin 1β (IL-1β), responsible for inflammation-induced neuronal death (Saijo et al., 2009; McMorrow and Murphy, 2011). In addition, Nurr1 induces the expression of neurotrophic factors, such as brain-derived neurotrophic factor (BDNF) (Barneda-Zahonero et al., 2012). In addition to its role in the CNS, Nurr1 has an active role in PD, as downregulated levels of gene expression were also found in peripheral blood obtained from PD patients with progressive loss of mDA neurons (Le et al., 2008; Liu et al., 2012; Montarolo et al., 2016).

Recently, it has been shown that Nurr1 also controls the expression of several nuclear-encoded mitochondrial genes involved in oxidative respiration, such as *SOD1*, Ts translation elongation factor (*TSFM*) and cytochrome *c* oxidase subunit 5B (*COX5B*), demonstrating an important role in sustaining respiratory function (Kadkhodaei et al., 2013). Therefore, we first analyzed Nurr1 gene expression levels in blood obtained from ALS patients in comparison with healthy controls (HC). To better understand its role in ALS, we then investigated the expression and function of Nurr1 in a murine model of ALS, the SOD1-G93A mouse.

## RESULTS

### Nurr1 mRNA is upregulated in the peripheral blood of ALS patients

**Fig. 1.**
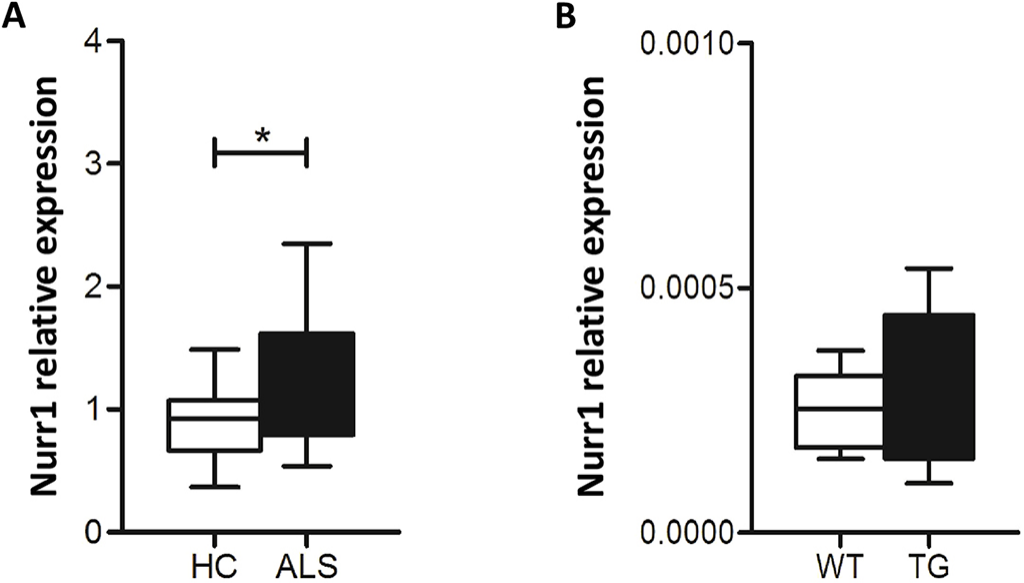
**Whole peripheral blood gene expression levels of Nurr1 in ALS patients and in SOD1-G93A mice.** (A) Comparison of gene expression levels of Nurr1 in 43 ALS patients and 41 HC. Nurr1 is upregulated in ALS patients compared with HC (Student's *t*-test, *P=0.01). Relative expression was calculated using the normalized comparative cycle threshold (Ct) method (2^-ΔCt). (B) Comparison of gene expression levels of Nurr1 in seven WT and eight TG mice. No differences were detected for Nurr1 in TG mice compared with HC (Student's *t*-test, *P*=0.69). Relative expression was calculated using the normalized comparative cycle threshold (Ct) method (2^-ΔCt).

Gene expression analysis of Nurr1 was performed on whole peripheral blood obtained from 43 ALS patients and 41 HC subjects; demographic and clinical characteristics are summarized in Table 1. The ALS group, comprising seven fALS and 36 sALS patients, showed higher Nurr1 mRNA levels compared with the HC group (Fig. 1A). Furthermore, there was a significant difference in age (Student's *t*-test, *P*=0.002; Table 1), but not in sex between ALS patients and HC (Fisher exact test, *P*=0.38; Table 1). To assess potential bias related to age, the correlation between age and gene expression levels of Nurr1 was evaluated in HC subjects and no significant results were highlighted, as previously reported by Montarolo et al. (2016) (Pearson correlation coefficient r=0.13, *P*=0.41). Also, correlation analyses between Nurr1 gene expression of ALS patients and age at the time of sampling (Pearson correlation coefficient r=−0.09, *P*=0.55) and age at disease onset (Pearson correlation coefficient r=−0.16, *P*=0.31) (Fig. S1A) did not highlight significant results. Similarly, there are no significant differences in Nurr1 expression between sexes in both HC (Student's *t*-test, *P*=0.53) and ALS patients (Mann–Whitney *U*-test, *P*=0.32) (Fig. S1B). The influence on Nurr1 expression level of the pharmacological treatment indicated in Table 1 was not assessed, owing to the small sample size of each group. Among fALS patients, four carried mutations in *C9Orf72*, two in *SOD1* and one in the *TARDBP* gene, whereas three sALS patients carried mutations in *C9Orf72*, *SOD1* and in the gene encoding optineurin (*OPTN*). We also reported Nurr1 expression levels for the different groups (Fig. S1C).

**Table 1.**
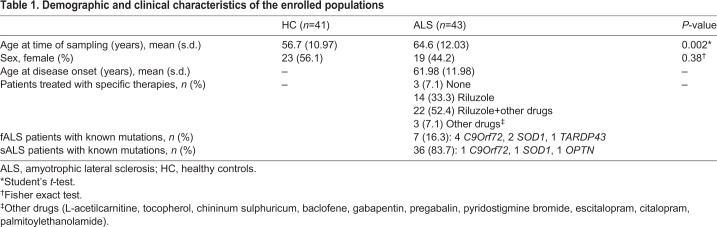
**Demographic and clinical characteristics of the enrolled populations**

### Nurr1 mRNA and protein are upregulated in the spinal cord of SOD1-G93A mice in the asymptomatic and early symptomatic phases of the disease

The high levels of Nurr1 expression in samples of peripheral blood from ALS patients suggested a potential role in ALS pathogenesis. To investigate the role of Nurr1 in ALS outcome, we used a transgenic mouse model of ALS, SOD1-G93A (TG). This model has a high copy number of the transgene and is one of the most commonly used in this area of research (Gurney et al., 1994). Male mice develop first symptoms of the disease at approximately 3 months of age and die about 4 weeks after disease onset. Behavioral tests such as rotarod and paw grip endurance (PaGE) were used to evaluate the appearance of the first motor deficits and to divide the animals into three experimental groups: (1) asymptomatic mice; (2) early symptomatic mice; and (3) late symptomatic mice (Boido et al., 2014). Age-matched wild-type (WT) male mice were used as controls. Female animals were excluded from the study as significant differences were observed between male and female TG animals, probably owing to an estrogen neuroprotective effect (Vercelli et al., 2008).

**Fig. 2.**
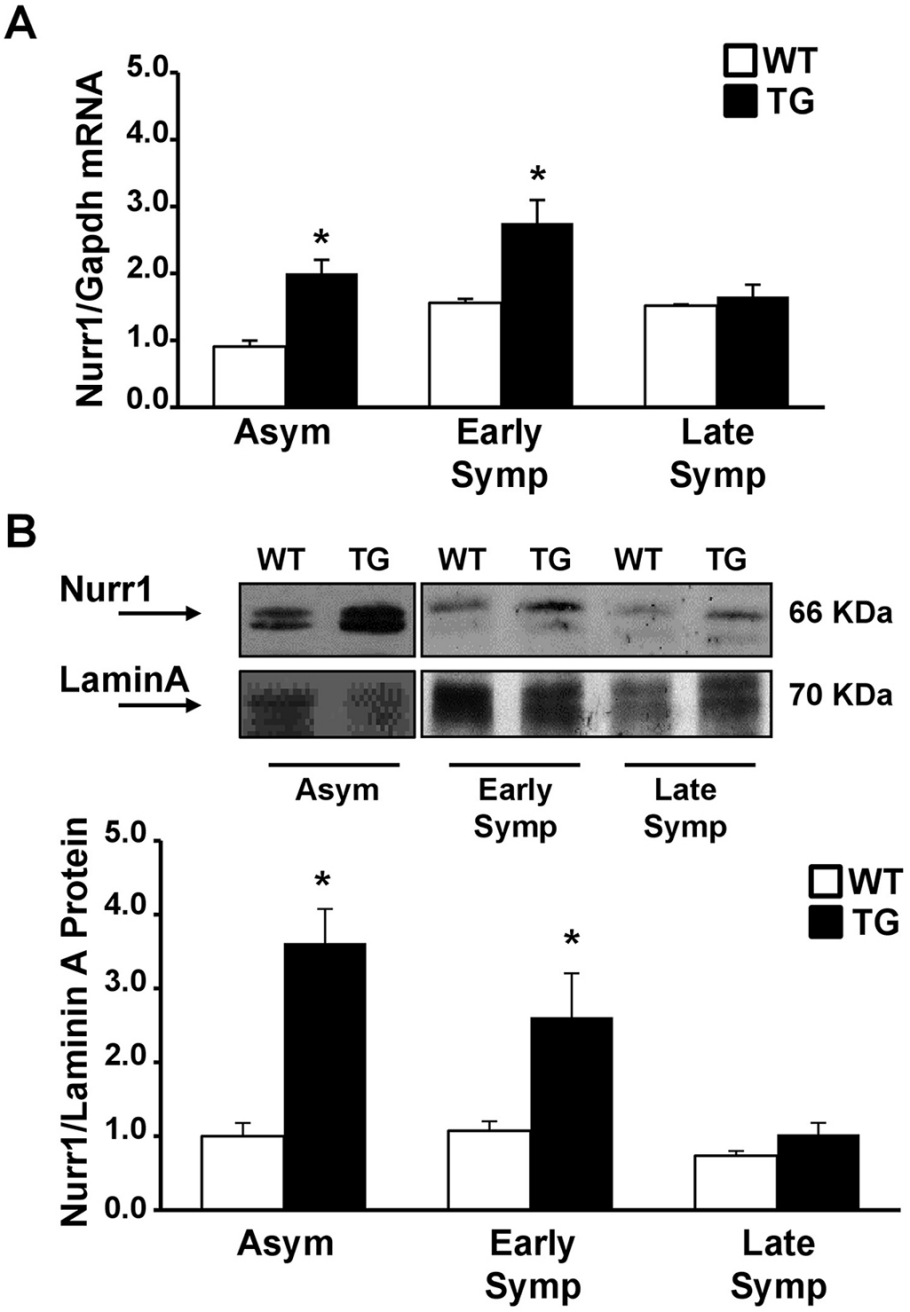
**Nurr1 mRNA and protein were upregulated in the spinal cord of asymptomatic and early symptomatic SOD1-G93A mice.** (A) mRNA expression levels of Nurr1 in the spinal cord of asymptomatic (Asym, *n*=11), early symptomatic (Early Symp, *n*=5) and late symptomatic (Late Symp, *n*=4) SOD1-G93A animals (TG, black columns), compared with age-matched WT mice (white columns). The *Gapdh* gene was used as endogenous control. (B) Representative western blot displaying the expression levels of Nurr1 protein in nuclear extracts from spinal cord of Asym (*n*=8), Early Symp (*n*=5) and Late Symp (*n*=3) TG and WT animals. The graph below the image reports the quantification of Nurr1, expressed as a ratio with the endogenous control lamin A. Each column represents the mean±s.e.m. Statistically significant differences among means were determined by two-way ANOVA followed by Bonferroni test. *P<0.05 TG versus respective WT.

First, we measured Nurr1 mRNA levels in the whole peripheral blood obtained from early and late symptomatic TG animals, considered as a single group. The analysis revealed no differences in Nurr1 expression levels between TG and age-matched WT mice (Fig. 1B). By contrast, on evaluating Nurr1 expression levels in the spinal cord of TG mice (Fig. 2A,B), we observed that Nurr1 mRNA was significantly upregulated (up to 2.2-fold) in the asymptomatic phase of the disease compared with respective controls. In the early symptomatic phase of the disease, Nurr1 mRNA content was still higher in TG mice than in WT animals, but to a slightly lesser extent (1.8-fold); in end-stage TG mice, Nurr1 mRNA levels were the same as for age-matched WT controls (Fig. 2A).

Nurr1 protein levels in nuclear extracts obtained from the spinal cord of TG mice confirmed the results obtained with mRNA. In particular, Nurr1 protein was strongly upregulated by 3.6- and 2.6-fold in the nuclei of asymptomatic and early symptomatic TG mice, respectively, compared with WT controls (Fig. 2B). By contrast, WT animals presented comparable levels of Nurr1, both at the mRNA and protein level, in all groups analyzed (Fig. 2A,B).

### Nurr1 is able to activate *Bdnf* expression and to prevent NFκB target gene activation in the asymptomatic phase of the disease of SOD1-G93A mice

We investigated the possible role of Nurr1 by comparing TG and WT spinal cord samples. As it has been demonstrated that the transcription factor Nurr1 can directly activate its target genes, such as *Bdnf* (Barneda-Zahonero et al., 2012), we performed a real-time PCR analysis for *Bdnf*. Furthermore, Nurr1 can dock on p65, the NFκB transactivating subunit, blocking the activation of its pro-inflammatory genes, such as *iNos* (Saijo et al., 2009; De Miranda et al., 2015). Therefore, to investigate whether Nurr1 was involved in the NFκB pathway, we measured *iNos* mRNA and performed chromatin immunoprecipitation assay following quantitative real-time PCR (ChIP-qPCR) on the *iNos* promoter using antibodies against Nurr1 or p65.

**Fig. 3.**
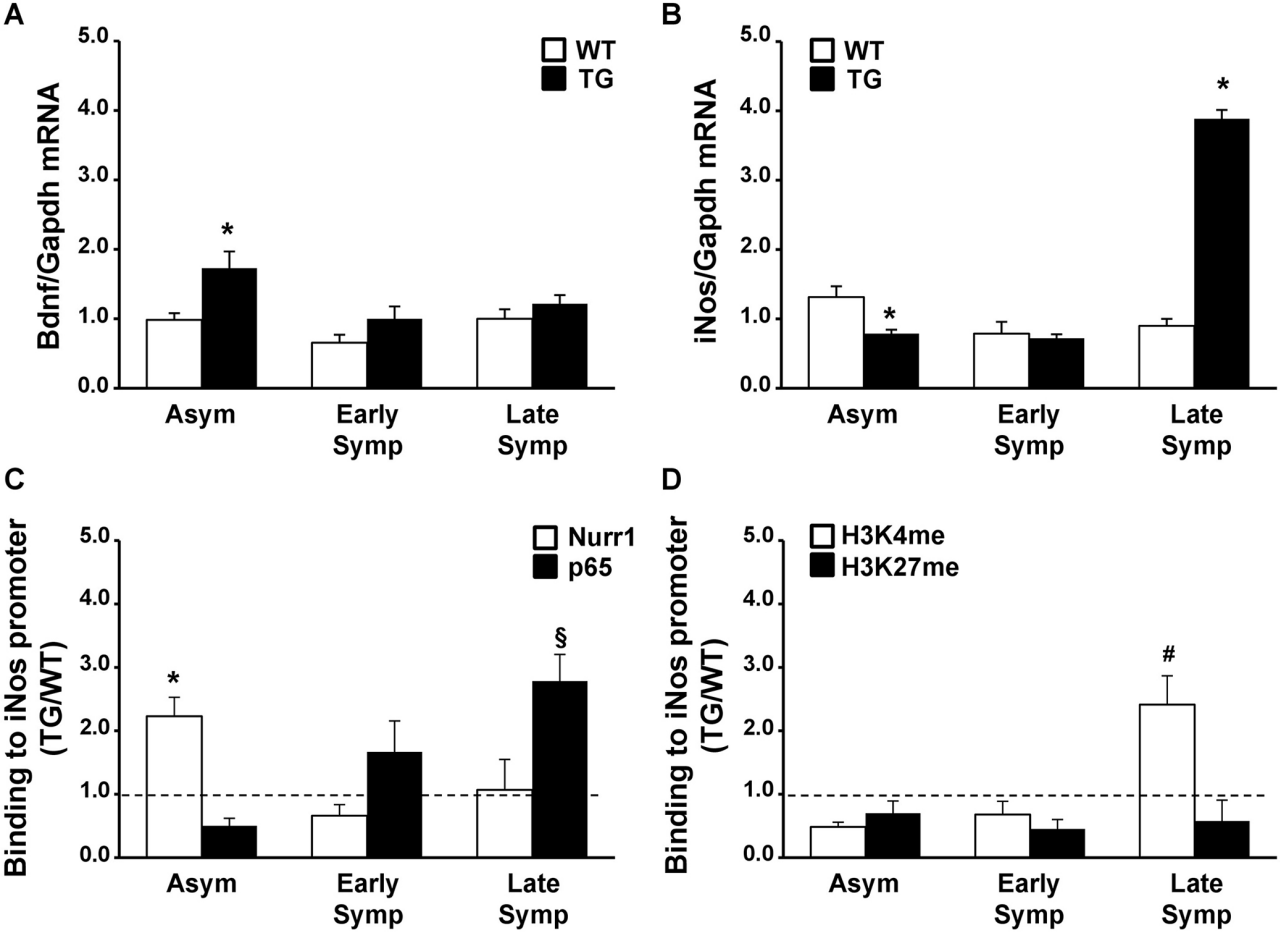
**Nurr1 activated *Bdnf* expression and repressed *iNos* transcriptional activation by docking with NFκB on the *iNos* promoter.** (A,B) mRNA expression levels of *Bdnf* (A) and *iNos* (B) in the spinal cord of asymptomatic (Asym, *n*=10), early symptomatic (Early Symp, *n*=5) and late symptomatic (Late Symp, *n*=3 for panel A and *n*=4 for panel B) SOD1-G93A animals (TG, black columns), compared with age-matched WT mice (white columns). The *Gapdh* gene was used as an endogenous control. Each column represents the mean±s.e.m. Statistically significant differences among means were determined by two-way ANOVA followed by Bonferroni test. *P<0.05 TG versus respective WT. (C,D) ChIP analysis of Nurr1 and p65 (C) and H3K4me and H3K27me (D) on the *iNos* promoter in the spinal cord of Asym, Early Symp and Late Symp TG and WT animals. The binding activity of each transcription factor was calculated as the percentage of total input of chromatin DNA and represented as the ratio between TG and age-matched WT animals. Each column represents the mean±s.e.m. (*n*=3). Statistically significant differences between means were determined by two-way ANOVA followed by Bonferroni test: *P<0.05 Nurr1 versus p65; ^§^*P*<0.05 p65 versus Nurr1; ^#^*P*<0.005 H3K4me versus H3K27me.

Our results showed a significant increase in *Bdnf* mRNA levels in the asymptomatic phases of the disease (Fig. 3A). In particular, a significant increase of 1.8-fold was observed in asymptomatic TG animals compared with WT. In early symptomatic mice, *Bdnf* levels were still higher than in respective controls, but not significantly (1.5-fold). In a late phase of the disease, *Bdnf* levels were comparable with those of respective WT controls.

By contrast, we observed a downregulation of *iNos* mRNA in the asymptomatic phases of the disease (up to 40%), as compared with age-matched WT controls; this downregulation was not seen in early symptomatic mice. Furthermore, in a late phase of the disease *iNos* mRNA was strongly upregulated up to 4.3-fold in TG animals compared with respective controls (Fig. 3B).

To assess whether *iNos* modulation depended on direct competition between Nurr1 and NFκB binding on its promoter, we performed ChIP-qPCR assay (Fig. 3C). In asymptomatic animals, Nurr1 binding, expressed as TG to WT ratio, was 2.2-fold higher in TG compared with age-matched WT animals. Interestingly, at this stage, Nurr1 binding was 4.6-fold higher than p65 binding at the *iNos* promoter. Furthermore, during disease progression Nurr1 binding decreased, and this was mirrored by a parallel increase in p65 binding. Specifically, in late symptomatic TG animals p65 binding was 2.8-fold higher than for WT, and the TG/WT ratio of p65 binding was 2.6-fold higher compared with Nurr1 (Fig. 3C). Finally, we performed ChIP-qPCR assay on the *iNos* promoter using antibody against trimethylation at Lys4 of histone H3 (H3K4me3) and trimethylation at Lys27 of histone H3 (H3K27me3), markers for active and repressive genes, respectively (Kim, et al., 2013). Our results showed that H3K4me3 enrichment increased 4.2-fold compared with H3K27me3, suggesting that the *iNos* promoter is active in accordance with increased *iNos* mRNA levels (Fig. 3D).

### Nurr1 protein is expressed in motor neurons and, to a lesser extent, in astrocytes of SOD1-G93A mice

To investigate in which CNS cell types Nurr1 was expressed, representative double immunofluorescence experiments were performed in spinal cord sections with antibodies raised against three markers: (1) the neurofilament H (SMI32), a specific marker for MNs; (2) the glial fibrillary acidic protein (GFAP), an intermediate filament protein expressed mainly by astrocytes in the CNS; and (3) CD68 protein, which is highly expressed by macrophages and activated microglia.

**Fig. 4.**
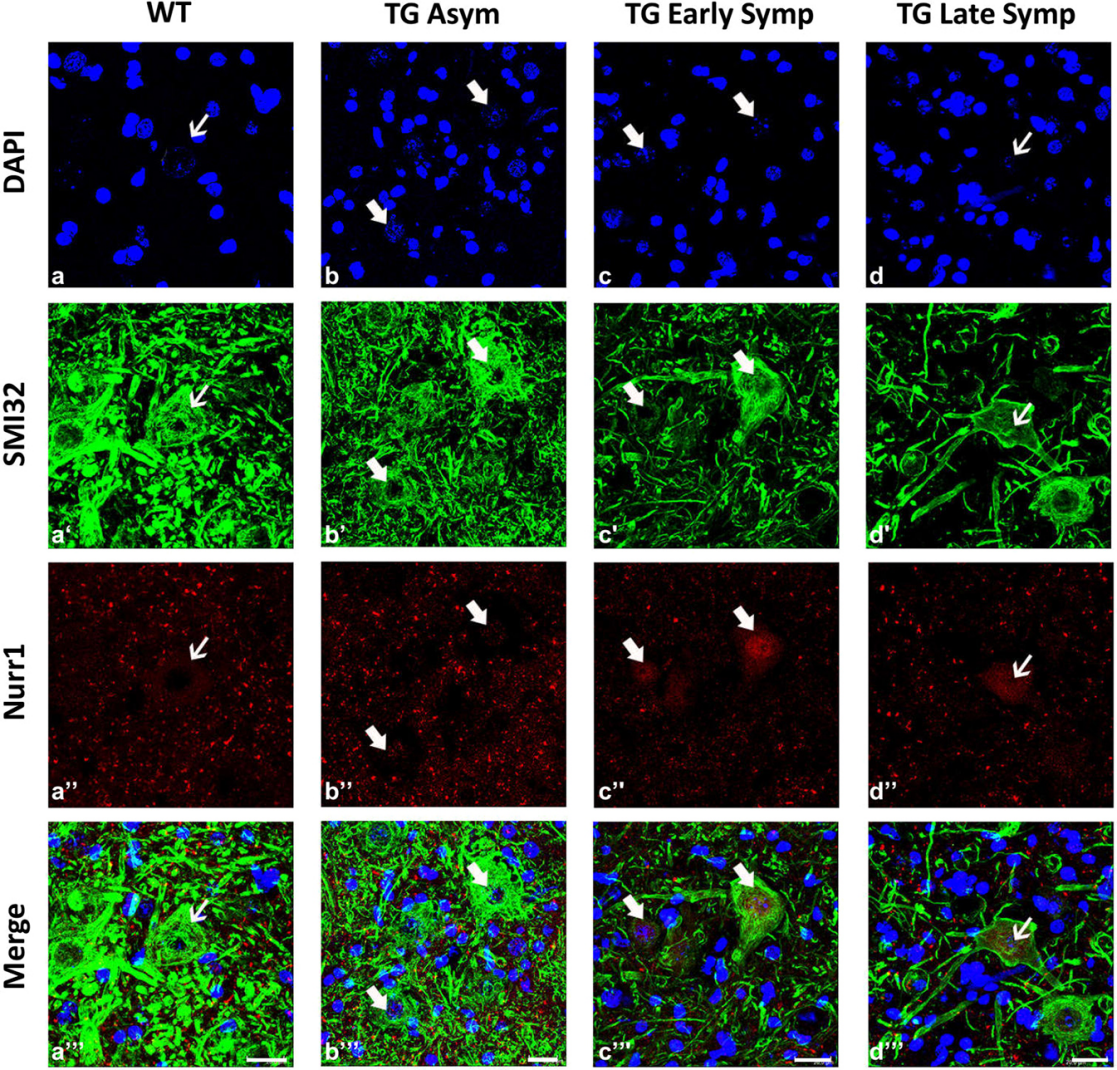
**Nurr1 protein is expressed in MNs of TG animals.** Representative confocal images showing the double-labeling of Nurr1 (red) and SMI32 (green) in lumbar spinal cord of WT animals (a-a′″), asymptomatic (Asym, b-b′″), early symptomatic (Early Symp, c-c′″; e-e′″) and late symptomatic (Late Symp, d-d′″) TG mice. Nuclei are labeled with DAPI (blue). Thin arrows show mainly cytoplasmic localization of Nurr1, whereas thick arrows indicate the nuclear localization of Nurr1 in MNs. Scale bars: 20 µm.

For the first time, we observed that Nurr1 is physiologically expressed in the cytoplasmic compartment of SMI32-positive cells of WT animals, indicating Nurr1 presence in MNs (Fig. 4, a″-a′″, thin arrows). Interestingly, in asymptomatic and early symptomatic TG mice, Nurr1 expression was mainly evident in the nuclear compartment of MNs (Fig. 4, b″-b′″,c″-c′″, thick arrows), in agreement with the upregulation in nuclear extract observed with western blot (WB) analysis. By contrast, in late symptomatic TG animals, Nurr1 immunostaining was still present only in MN cytoplasm (Fig. 4, d″-d′″, thin arrows).

Furthermore, as neuroinflammation is a characteristic of ALS (see Figs S2 and S3, in agreement with Ilieva et al., 2009; Boido et al., 2014; Anzilotti et al., 2018), and because Nurr1 is reported to have an anti-inflammatory role in astrocytes and microglial cells (Saijo et al., 2009), we investigated its expression in GFAP- and CD68-positive cells.

**Fig. 5.**
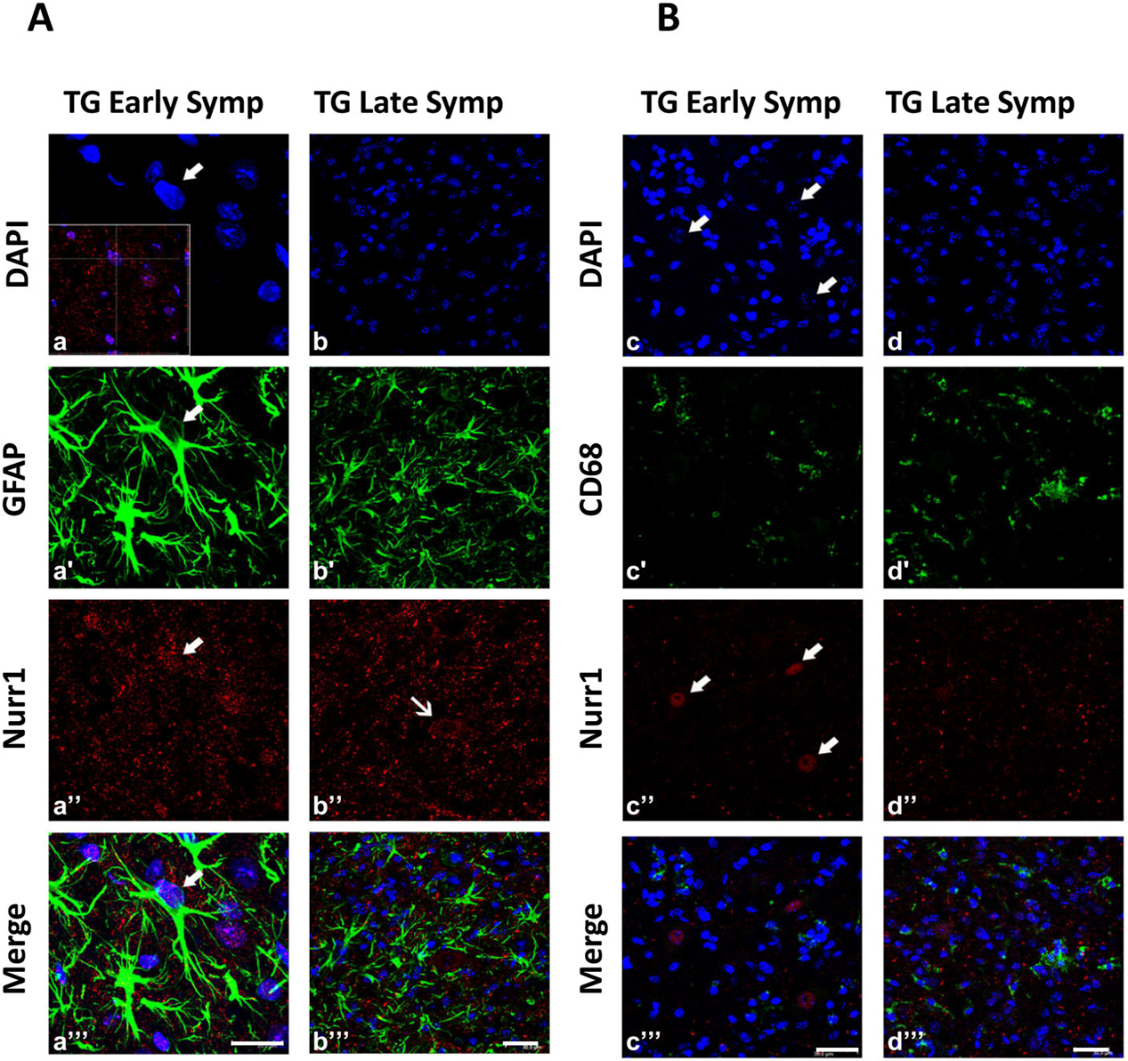
**Nurr1 protein is expressed in astrocytes of TG animals.** (A,B) Representative confocal images showing the double-labeling of Nurr1 (red) and GFAP (green; A) or CD68 (green; B) in lumbar spinal cord of early symptomatic (Early Symp, a-a′″; c-c′″) and late symptomatic (Late Symp, b-b′″; d-d′″) TG mice. Nuclei are labeled with DAPI (blue). Thin arrows show mainly cytoplasmic localization of Nurr1, whereas thick arrows indicate nuclear localization of Nurr1 in astrocytes. In the inset in a, rotations along the *x*- and *y*-axes show the superposition of the two colors on the *z*-axis. In b″ (TG, Late Symp), it is evident that the astrocytes are not labeled, but they surround a plausible Nurr1-positive MN (Nurr1 expression at the cytoplasmic level, thin arrow). B demonstrates the absence of Nurr1-positive microglial cells, further confirming its expression in plausible MNs (thick arrows). Scale bars: 20 µm (a-a′″) and 30 µm (b-b′″, c-c′″ and d-d′″).

Double immunofluorescence staining with GFAP and Nurr1 showed the Nurr1 immunosignal to be present in the nuclear compartment of rare astrocyte cells at early stages of the disease (Fig. 5A, a″-a′″, thick arrows), but it was absent in the late symptomatic phase (Fig. 5A, b″,b′″).

By contrast, double immunofluorescence staining with CD68 and Nurr1 did not highlight Nurr1 expression in CD68-positive cells (Fig. 5B, c″,c′″,d″,d′″).

## DISCUSSION

In this paper, we investigated the expression of the transcription factor Nurr1 in blood obtained from ALS patients and in the blood/spinal cord of a murine model of ALS, the SOD1-G93A mouse. Nurr1 is known to have an important role in the maturation of mDA neurons (Kadkhodaei et al., 2009), linking its deficiency mainly to PD. In fact, several human mutations in the gene encoding for Nurr1 protein, *NR4A2*, are associated with late-onset familial PD (Le et al., 2003) and the SNPsrs35479735 (insertion/deletion of a G) seems to be a significant risk factor for the development of PD (Liu et al., 2017; Ruiz-Sánchez et al., 2017). Furthermore, Nurr1 gene expression is downregulated in the blood of PD patients with progressive loss of DA neurons (Le et al., 2008; Liu et al., 2012; Montarolo et al., 2016). Moreover, conditional ablation of Nurr1 in adult mature mDA neurons in mice resulted in the downregulation of several genes involved in oxidative respiration, and particularly of SOD1 (Kadkhodaei et al., 2013). As SOD1 is a key enzyme in ALS disease (Renton et al., 2014; Chia et al., 2018), these observations prompted us to explore the functional role of Nurr1 in ALS pathology.

In the whole blood obtained from ALS patients, we noted a significant increase in mRNA levels of Nurr1 compared with HC. Nurr1 has a central role in immune homeostasis, where it regulates the induction, maintenance and suppressor functions of regulatory T cells (Tregs). Specifically, it represses aberrant Th1 induction through transcriptional activation of the master transcription factor of Treg cells, the forkhead transcription factor (*Foxp3*), and inhibits production of the cytokines interferon γ (IFNγ) and interleukin-17 (IL-17) (Sekiya et al., 2011, 2013). Nurr1 mRNA upregulation in the blood of ALS patients suggested an additional important role for this transcription factor in ALS, in which immune cells are able to exert either a detrimental or a protective action on MN survival (Chiu et al., 2008; Hooten et al., 2015). Indeed, there is convincing evidence to suggest that ALS is a systemic disorder characterized by peripheral immune alterations (McCauley and Baloh, 2019). Autopsy of ALS patients showed modifications in the frequency of circulating immune cell populations and in cytokine expression (Zhang et al., 2005; McCombe and Henderson, 2011; Murdock et al., 2017). However, the peripheral mechanism that crucially contributes to the ALS disease process, and whether or not it represents a consequence or has a causative role, is still unclear. Nurr1 upregulation could be driven by a response to tissue damage in the brain, initially sensed by resident glial cells then amplified and propagated by the peripheral immune cells or by an intrinsically altered peripheral immune system in ALS patients. Indeed, Nurr1 expression has been reported in dendritic cells (Saini et al., 2016), T cells (Sekiya et al., 2011; Won and Hwang, 2016) and in macrophages (Bonta et al., 2006).

To shed light on the role of Nurr1 in ALS pathology, we investigated Nurr1 expression and its mechanism of action in the SOD1-G93A mouse, one of the most commonly used ALS murine models. Specifically, we assessed Nurr1 expression levels in the blood of early and late symptomatic animals, considering them a unique group to better represent ALS patient sampling. However, the blood of TG mice did not show a significant increase in Nurr1 expression compared with age-matched WT animals; this observation was in contrast to the increase seen in ALS patients. This discrepancy is probably a consequence of the small sample size and/or because the phases of the disease in human ALS and the murine model are not parallel. Furthermore, our mouse model carried a mutation in the SOD1 gene, which represents only 7% of our ALS patient cohort.

By contrast, in the spinal cord of TG mice our data revealed that Nurr1 was strongly upregulated in the early phases of the disease in TG mice; indeed, (1) it translocated into the nuclei of MNs and (to a lesser extent) astrocytes; (2) it induced neurotrophic factor *Bdnf* expression; and (3) it inhibited *iNos* expression by docking with NFκB on the *iNos* promoter. These observations have been made using reverse transcription PCR (RT-PCR), WB analysis, ChIP-qPCR assay and immunofluorescence staining. Concerning immunofluorescence experiments, the Nurr1 antibody is most commonly used in immunocytochemistry (Zhou et al., 2010; Lee et al., 2012; Alvarez-Castelao et al., 2013), as on tissue samples it tends to give an undesired noisy background (Baron et al., 2012; Garcia-Perez et al., 2013) despite the antigen retrieval passage. Nevertheless, we are quite confident about the labeling specificity seen here, as it reflects the molecular trend observed with RT-PCR and WB assays.

For the first time, our results highlight Nurr1 expression in spinal cord MNs. Indeed, Nurr1 was physiologically present in the cytoplasmic compartment of MNs from WT animals, suggesting a potential role for this transcription factor in the survival of cholinergic neurons, which are affected in this neurodegenerative disease. Notably, very early in disease progression (in asymptomatic and early symptomatic phases) Nurr1 was upregulated at the transcriptional level, as suggested by RT-PCR analysis. Following activation, Nurr1 was translocated to the nucleus, as indicated by its increased levels in nuclear extracts of TG mice; Nurr1 was found mainly in MNs and rarely in astrocytes, as indicated by immunofluorescence experiments. Within the nuclei of asymptomatic animals, Nurr1 was able to activate *Bdnf*, its target gene, and to repress the transcription of *iNos*. Indeed, Nurr1 upregulation in the asymptomatic phase of the disease was accompanied by an increase in *Bdnf* mRNA and a reduction of *iNos* mRNA, as demonstrated by RT-PCR assay. Repression of *iNos* was probably the result of direct competition between Nurr1 and NFκB on the *iNos* promoter, as indicated by ChIP-qPCR assay, in agreement with the mechanism of action proposed by Sajio and coworkers for PD (Saijo et al., 2009). In the early symptomatic phase, however, even if Nurr1 levels in the nuclear compartment of TG mice were higher than those in age-matched WT, it was not able to activate *Bdnf* or repress *iNos*. Finally, in late symptomatic animals, when Nurr1 was no longer upregulated at the transcriptional level and apparently absent from nuclear compartments, we observed a strong binding of NFκB to the *iNos* promoter and an enrichment of H3K4me3, indicating that the gene was active in accordance with the observed increase in mRNA levels.

Therefore, we hypothesized that Nurr1 is not directly involved in the development of ALS pathology, but instead is likely to act as an endogenous means to delay the pathogenetic mechanisms. In particular, Nurr1 could be activated before symptom onset, as an endogenous neuroprotective mechanism with an anti-inflammatory role, although not sufficient to reverse disease progression. Preventing the final downregulation of Nurr1 and exploiting its ability to activate *Bdnf* and to repress *iNos* in the late stages of the disease could represent a useful therapeutic approach.

Nurr1 has recently emerged as having a key role in the mediation of cell-type specific inflammatory responses in several diseases, such as cancer, immune alterations, metabolic, cardiovascular and neurological diseases (Rodríguez-Calvo et al., 2017; Safe et al., 2016). In particular, Saijo and coworkers (Saijo et al., 2009) demonstrated Nurr1 to have an important anti-inflammatory role following microglial activation induced by LPS injection, limiting the production of neurotoxic mediators by glial cells and protecting DA neurons from inflammation. Furthermore, a neuroprotective effect of Nurr1 was demonstrated in the multiple sclerosis (MS) murine model, represented by experimental autoimmune encephalomyelitis (EAE). Indeed, it has been demonstrated that a preventive treatment with the Nurr1 activator isoxazolopyridinone 7e delays onset and reduces the incidence and severity of EAE, reducing inflammation in the spinal cord of treated mice probably through an NFκB-dependent mechanism (Montarolo et al., 2014).

Interestingly, Nurr1 is able to protect hippocampal neurons within the CA1 field following kainic acid insult in mice (Volakakis et al., 2010) and also emerged as a mediator of CREB-dependent neuroprotection in mouse embryonic stem cell derived neurons (Volakakis et al., 2010). By contrast, Nurr1, and NR4A family members in general, have also been described as pro-inflammatory factors with controversial results in several disease models (Rodríguez-Calvo et al., 2017).

### Conclusions

Collectively, our results demonstrate for the first time that Nurr1 mRNA is upregulated in blood samples of ALS patients; therefore, we speculate that it could be considered a biomarker candidate for ALS. However, further studies will be necessary to confirm this aspect and to determine the specific role of Nurr1 in the peripheral immune system during disease progression.

In addition, we demonstrate in a mouse model of ALS that Nurr1 is activated in the early symptomatic phase of the disease, probably as a neuroprotective endogenous mechanism. This observation is in agreement with the current hypothesis supporting an initial activation of glial cells aimed at sustaining MN viability through neurotrophic factors (IGF-1), anti-inflammatory interleukins (IL-4, IL-10) and cytokine secretion (Chiu et al., 2008; Henkel et al., 2009; Murdock et al., 2015; Hooten et al., 2015). Later on during disease progression, we observed the inactivation of Nurr1: it was no longer upregulated at the mRNA level, was absent from the nuclear compartment and was unable to dock with NFκB on the *iNos* promoter. Therefore, we speculate that Nurr1 might represent a promising target for ALS therapy, as neuroinflammation is a relatively unexplored field that can modify the course of ALS disease. Future therapeutics aimed at augmenting the anti-inflammatory effect through Nurr1 activation could mitigate the toxic environment, modulate neuroinflammation and foster the MN repair process, having a positive effect on ALS treatment.

## MATERIALS AND METHODS

### Enrolled subjects

A total of 43 patients affected by ALS and 41 healthy controls (HC) were enrolled in the current study. ALS patients were followed and clinically monitored by neurologists of the ALS Expert Center (CRESLA), ‘Città della Scienza e della Salute’, University Hospital, Turin. Most of the ALS patients received disease-specific drugs at the time of blood sampling, such as riluzole alone or combined with symptomatic therapies. HC, recruited from volunteers, were asked to complete a health questionnaire to exclude any acute or chronic inflammatory and neurological disease. All subjects enrolled in the study were of Caucasian origin. Demographic and clinical features of patients and HC are summarized in Table 1.

This study was approved by Piedmont and San Luigi University Hospital Ethical Committee (N°80/2011, 25 May 2011) and was conducted in accordance with the ethical standards laid down in the 1964 Helsinki declaration and its later amendments. Written informed consent was obtained from all individual participants included in the study at the time of blood drawing.

### Animal care and use

Experiments were performed on male transgenic mice B6SJL-TgN(SOD1G93A)1Gur overexpressing human SOD1, containing the Gly93Ala mutation (The Jackson Laboratory, stock number 002726); these mice have high transgene copy number, as reported in the datasheet. The colony was derived by breeding male transgenic (TG) mice with naive (B6xSJL/J)F1 females (WT) (Janvier SAS). Overall, 35 WT and 35 TG mice housed under diurnal lighting conditions (12 h darkness/light) were used. All experimental procedures on live animals were carried out in strict accordance with the European Communities Council Directive 86/609/EEC (24 November 1986) Italian Ministry of Health and University of Turin institutional guidelines on animal welfare (law 116/92 on care and protection of living animals undergoing experimental or other scientific procedures; authorization number 17/2010-B, 30 June 2010 and 367/2016-PR); additionally, an ad hoc Ethical Committee of the University of Turin approved this study. All efforts were made to minimize the number of animals used and their suffering. Transgenic mice were identified by PCR according to The Jackson Laboratory's genotyping protocol.

### Genotyping mice

DNA was extracted from the mouse tail as previously described (Sirabella et al., 2018). PCR was performed on the extracted DNA to evaluate the presence of the human transgene superoxide dismutase 1 (*hSOD1*) gene: these mice were referred to as TG. Two primers were used: hSOD1 fwd 5′-CATCAGCCCTAATCCATCTGA-3′ and hSOD1 rev 5′-CGCGACTAACAATCAAAGTGA-3′.

### Behavioral tests

To identify symptom onset and to follow disease progression, TG mice underwent specific behavioral tests: rotarod and PaGE tests were performed by a trained blind observer, as previously reported (Valsecchi et al., 2013; Boido et al., 2014). The tests started from postnatal day 60 (P60), a fully asymptomatic phase of the disease. The first 2 weeks of tests were considered as training for the animals. The tests were performed twice a week. The body weight was also monitored during the whole period of observation. Briefly, for the rotarod test we measured the time animals could remain on the rotating cylinder in a 7650 accelerating model of a rotarod apparatus (Ugo Basile, Italy). Each animal was given three trials. The arbitrary cut-off time was 300 s, and the accelerated speed went from 4 to 32 rpm. For PaGE tests, the animal was placed on the wire lid of a conventional housing cage: the lid was gently shaken to prompt the mouse to hold onto the grid before it was swiftly turned upside down. Grip score was measured as the length of time that the mouse was able to hang on to the grid. The arbitrary cut-off time was 90 s.

This G93A stock of mice has a high transgene number and shows the first symptoms of disease at approximately 3 months of age, with a rapid progression of the disease in 1 month. We considered three groups of animals: (1) asymptomatic, mice between 2 and 3 months of age that do not display any motor performance deficit; (2) early symptomatic, animals that displayed decreased motor behavioral performance in two consecutive testing sessions, approximately at 3.5 months of age; and (3) late symptomatic, mice of 4 and 4.5 months of age with seriously compromised motor conditions, as previously reported.

### RT-PCR analysis

#### ALS patients

Peripheral whole blood samples from ALS patients and HC were collected into Tempus Blood RNA Tubes (Thermo Fisher Scientific) and stored at −80°C until use. Total RNA was automatically extracted using the Maxwell RSC Station and products (Promega), following the manufacturer's instructions, and was reverse-transcribed at a final concentration of 20 ng/μl using the RT High Capacity Transcription Kit following manufacturer's instructions (Life Technologies).

#### Mice

Mice were deeply anaesthetized with 3% isoflurane vaporized in O_2_/N_2_O 50:50 and sacrificed. The blood was rapidly collected in Tempus Blood RNA Tubes (Thermo Fisher Scientific) and stored at −80°C until use. Total RNA was automatically extracted using the Maxwell RSC Station and products (Promega), following the manufacturer's instructions, and was reverse-transcribed at a final concentration of 20 ng/μl (Thermo Fisher Scientific) using the RT High Capacity Transcription Kit following manufacturer's instructions (Life Technologies). The spinal cord was rapidly removed and immediately frozen on dry ice and stored at −80°C until use. Total RNA was extracted with Trizol, following supplier's instructions (Life Technologies) and cDNA was synthesized using 2 µg of total RNA with the High Capacity Transcription Kit following manufacturer's instruction (Life Technologies), as previously reported (Sisalli et al., 2014; Formisano et al., 2015).

Gene expression analysis was performed by RT-PCR using Applied Biosystems' TaqMan gene expression products (Life Technologies). For HC and ALS patients, primers from Applied Biosystems' TaqMan Assay-on-demand^TM^ gene expression products were used: glyceraldehyde-3-phosphate dehydrogenase (*GAPDH*; Hs99999905_m1) and Nurr1 (Hs00428691_m1) (Life Technologies), as previously reported (Montarolo et al., 2015). For mice, Applied Biosystems' TaqMan gene expression assay Nurr1 (TaqMan ID: Mm00443060_m1) and *Gapdh* (ID: Mm99999915_g1) were used as previously reported (Valsecchi et al., 2015). Expression levels of target genes were calculated by the normalized comparative cycle threshold (Ct) method (2^−ΔΔCt), using *GAPDH* as reference gene and the Universal Human Reference RNA (Stratagene) as calibrator for human samples. For blood murine samples, expression levels of target genes were calculated by the normalized comparative cycle threshold (Ct) method (2^−ΔCt), using *Gapdh* as reference gene.

### WB analysis

Nuclear and cytoplasmic extracts were obtained as previously described (Guglielmotto et al., 2014; Piras et al., 2017). Briefly, samples (spinal cord) were first washed with cold phosphate-buffered saline (PBS) and then homogenated with a 28-gauge needle syringe in ice-cold lysis buffer (10 mM HEPES pH 7.9, 10 mM KCl, 1.5 mM MgCl_2_, 0.1 mM EGTA, 0.2 mM PMSF, 10 mM NaF, 1 mM Na_3_VO_4_, 0.5 g/ml apronitin, 1 g/ml leupeptin, 1 g/ml pepstatin). Tissues were allowed to swell on ice for 10 min, vortexed and collected by centrifugation. The supernatant was discarded and the pellet dissolved in ice-cold buffer (20 mM HEPES pH 7.9, 400 mM NaCl, 1.5 mM MgCl_2_, 0.1 mM EGTA, 0.2 mM PMSF, 10 mM NaF, 1 mM Na_3_VO_4_, 0.5 g/ml apronitin, 1 g/ml leupeptin, 1 g/ml pepstatin) and incubated on ice for 30 min for high salt extraction. Cellular debris was removed by centrifugation and the supernatant fraction stored at −80°C.

Protein concentration was determined using the Bio-Rad protein assay. To detect the proteins of interest, specific antibodies were used: anti-Nurr1 (mouse monoclonal antibody, 1:750; Santa Cruz Biotechnology) and anti-lamin A (rabbit polyclonal, 1:1000; Swant). Immunoreaction was revealed using anti-mouse and anti-rabbit immunoglobulin G conjugated to peroxidase 1:2000 (GE Healthcare) by the ECL reagent (GE Healthcare). The optical density of the bands was determined by Chemi Doc Imaging System (Bio-Rad) and normalized to the optical density of lamin A.

### Chromatin immunoprecipitation assay

The chromatin immunoprecipitation assay and qPCR quantification were performed as previously described (Valsecchi et al., 2013; Guida et al., 2017, 2018; Iannello et al., 2019). Specifically, tissues were crosslinked with 1% formaldehyde in PBS for 10 min at 37°C. The reaction was stopped by adding glycine to a final concentration of 125 mM at room temperature (RT). Crosslinked spinal cords were washed three times in cold PBS containing proteinase inhibitors and then collected in 1 ml cell lysis buffer (5 mM PIPES pH 8, 85 mM KCl and 0.5% NP-40). After 10 min incubation on ice, nuclei were collected by centrifugation and lysed with 400 µl of nuclei lysis buffer (50 mM Tris-HCl pH 8, 10 mM EDTA and 1% SDS). The lysates were incubated on ice for 10 min and then sonicated 20 times for 20 s at 30% amplitude with SonoPlus HD2070 sonicator (Bandelin). A small portion of sonicated chromatin (25 µl) was used to verify that the average size of DNA fragments was in the range 250–500 bp. For each immunoprecipitation, a 1 µg sample of sheared chromatin was diluted in IP buffer (16.7 mM Tris-HCl pH 8, 167 mM NaCl, 1.2 mM EDTA, 0.01% SDS, 1.1% Triton X-100) and incubated with 0.5 µg of antibodies against Nurr1 and NFkB p65 (cat. numbers sc-990 and sc-372, respectively; Santa Cruz Biotechnology), histones H3K4me3 and H3K27me3 (cat. numbers 39915 and 39155, respectively; Active Motif) in a BSA pretreated 96-well dish at 4°C overnight on an orbital shaker. Samples with IgG antibody (cat. number sc-2027; Santa Cruz Biotechnology) were run in parallel as negative controls. The following day, 30 µl of 50% Protein A Sepharose™ 4 Fast Flow (GE Healthcare) slurry was added and incubated for 2 h at 4°C to capture the immune complexes. Proteins and DNA not specifically associated with the beads were removed by sequentially washing with low-salt buffer (0.1% SDS, 1% Triton X-100, 2 mM EDTA, 20 mM Tris-HCl pH 8 and 150 mM NaCl), high-salt buffer (0.1% SDS, 1% Triton X-100, 2 mM EDTA, 20 mM Tris-HCl, pH 8 and 500 mM NaCl), LiCl washing buffer (0.25 M LiCl; 1% deoxycholate sodium salt, 1 mM EDTA, 10 mM Tris-HCl pH 8 and 1% NP-40) and twice with Tris-EDTA buffer (10 mM TrisHCl pH 8, 1 mM EDTA) at 4°C for 5 min each wash. The immunoprecipitated DNA-protein complexes were purified using 10% Chelex^®^ 100 Resin (Bio-Rad) for 10 min at 95°C. Proteins were digested by incubating each sample with 20 μg of proteinase K (Thermo Fisher Scientific) for 30 min at 55°C and then 10 min at 95°C to obtain proteinase K inactivation, thus achieving DNA purification.

Quantification of ChIP-enriched DNA was performed by real-time PCR using iTaq Universal SYBR Green Supermix (Bio-Rad). The enrichment of target sequence in the immunoprecipitated samples was normalized on input samples (1% of total chromatin used per immunoprecipitation) and expressed as the ratio between WT and TG binding enrichment on *iNos* promoter for each transcription factor. Custom ChIP primers were employed: *iNos* promoter forward 5′-ATGCCATGTGTGAAAATTCC-3′ and reverse 5′-TGGGCTAGCCTGGTCTACAG-3′. Samples were amplified simultaneously in triplicate in one assay run.

### Immunofluorescence

Immunostaining procedures on spinal cord sections were performed as previously described (Boido et al., 2014, 2018). Briefly, animals (TG *n*=3, WT *n*=3 for each phase analyzed) were deeply anesthetized by gaseous anesthesia (3% isoflurane vaporized in O_2_/N_2_O 50:50) to undergo intracardiac perfusion with 4% paraformaldehyde (PFA) pH 7.4. The lumbar spinal cords were removed and post-fixed in PFA for 2 h at 4°C. Samples were transferred overnight into 30% sucrose in 0.1 M phosphate buffer at 4°C for cryoprotection, embedded in cryostat medium (Killik; Bio-Optica) and cut on the cryostat (Microm HM 550) in serial transverse 14 μm-thick sections. Sections were kept in PBS at 4°C or mounted onto gelatin-coated slides to be processed for immunostaining. Unspecific binding sites were blocked for 30 min at room temperature with 2% Triton X-100 and 10% normal donkey serum (Sigma-Aldrich) in PBS (pH 7.4). Sections were then incubated in the same solution with the following primary antibodies at 4°C overnight: 1:100 polyclonal rabbit anti-Nurr1 (cat. number sc991; Santa Cruz Biotechnology); 1:1000 monoclonal mouse anti-neurofilament H non-phosphorylated (SMI 32R; cat. number 14974402; Covance); 1:1000 monoclonal mouse anti-glial fibrillary acidic protein (GFAP; cat. number ab190288; Abcam); 1:1000 polyclonal rabbit anti-IBA1 (cat number 019-19741; Wako Chemicals); 1:1000 monoclonal rat anti-mouse CD68 (cat. number MCA1957; Bio-Rad). The next day sections were washed in PBS and incubated in 1:200 cyanine 3-conjugated anti-rabbit, Alexa Fluor^®^ 488 anti-mouse (1:200; respectively cat. numbers 711-165-152 and 715-546-150; Jackson ImmunoResearch) or anti-rat (1:200; cat. number ab150153; Abcam) secondary antibodies, depending on the primary antibodies used. Sections were then examined with a Leica TCS SP5 confocal laser-scanning microscope light. Photomicrographs were eventually manipulated with autocontrast enhancement by Photoshop CS2 software.

### Statistical analysis

Regarding ALS patients, continuous data are presented as medians and ranges and discrete data are given as counts and percentages. Chi-square tests were performed to compare groups of categorical data. Student's *t*-test or Mann–Whitney *U*-test were used to compare continuous data as appropriate. The correlation between Nurr1 gene expression levels and clinical and demographical variables was assessed by Pearson correlations and fitting linear models. In particular, we considered (Table 1): (1) sex and age at sampling for all the groups; (2) the age at disease onset. Data obtained from mice were expressed as mean±s.e. (s.e.m.). Statistically significant differences among means and/or ratios were determined by two-way ANOVA test followed by Bonferroni test. Statistical significance was considered at *P*<0.05. All analyses were carried out using R version 3.02 and Prism 5 software (GraphPad Software).

## Supplementary Material

Supplementary information
