# Supplementary material for: The transcription factor Nurr1 is upregulated in amyotrophic lateral sclerosis patients and SOD1-G93A mice
Source: Dis Model Mech. 2020 May 15;13(5):dmm043513. doi: 10.1242/dmm.043513 (PMC7240304; doi:10.1242/dmm.043513)
Supplement: Supplementary information [file dmm-13-043513-s1.pdf]

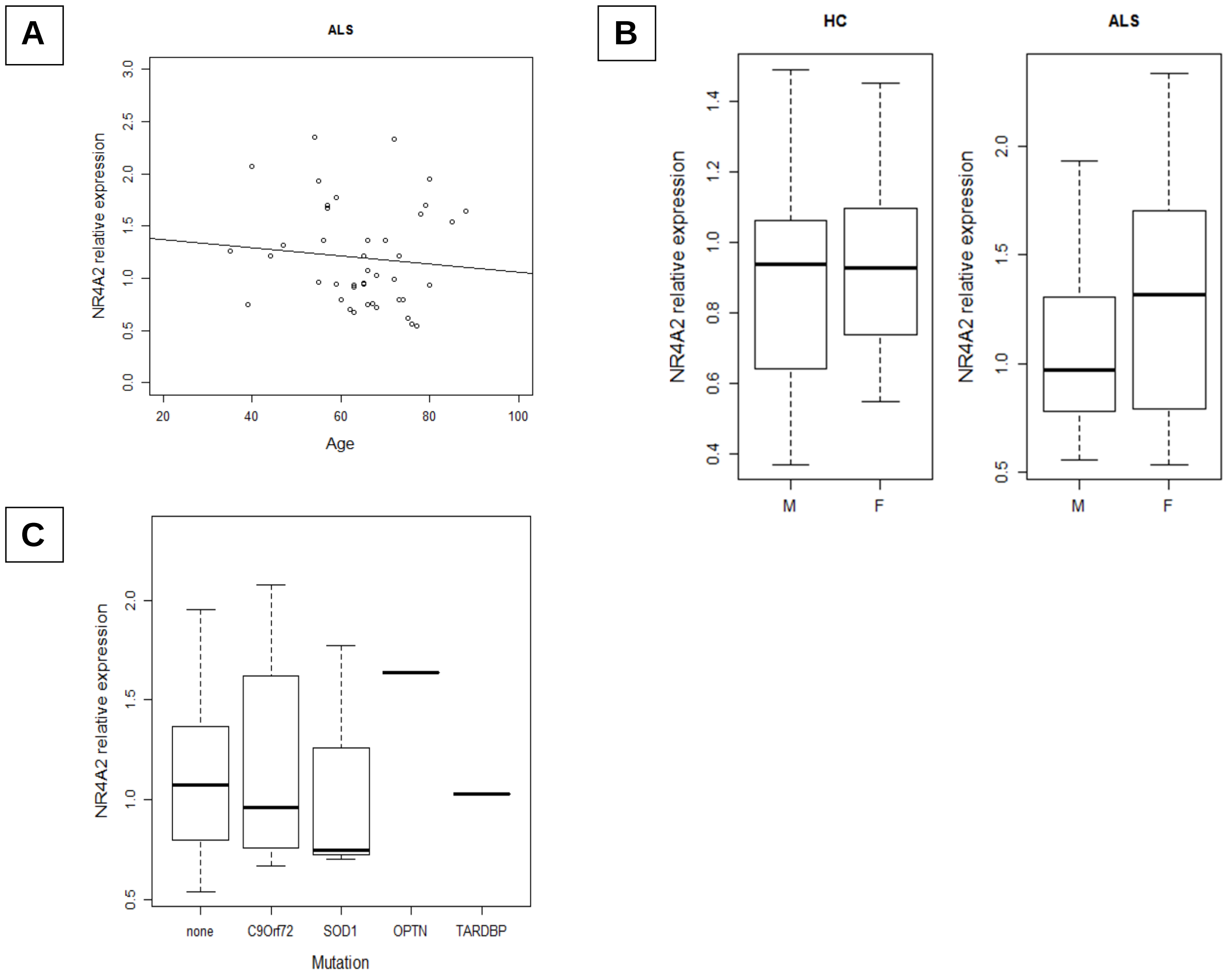

Figure S1. Correlation between Nurr1 expression levels and age, gender and mutations in ALS patients.

A, there is no correlation between Nurr1 expression levels and age of ALS patients (Pearson correlation coefficient r=-0.09, p=0.55). **B**, there are no significant differences in Nurr1 expression between males (M) and females (F) in both HC (t-test, p=0.53) and ALS patients (Mann-Whitney U test, p=0.32). **C**, Nurr1 expression levels for the different mutations identified in fALS and sALS patients. No association between Nurr1 level and the mutations identified was investigated due to the small sample size of each group.

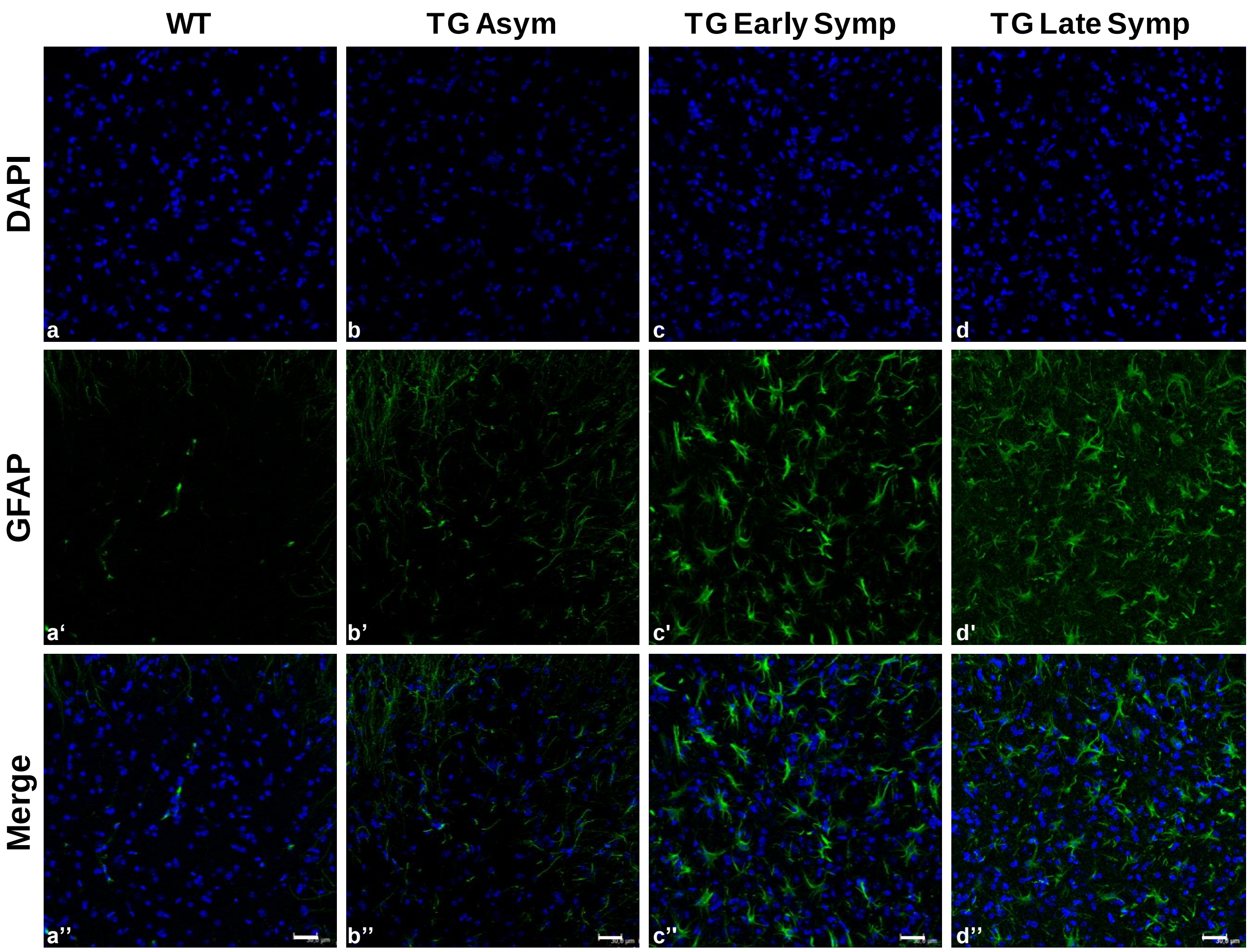

Figure S2. Astrogliosis activation analysis.

Representative confocal images showing reactive astrogliosis in terms of GFAP-labeling (green) in the lumbar spinal cord of WT animals (a-a'''), and Asym (b-b'''), Early Symp (c-c''') and Late Symp (d-d''') TG mice. Nuclei are labelled with DAPI (blue). Scale bar=30 μm.

Disease Models & Mechanisms • Supplementary information

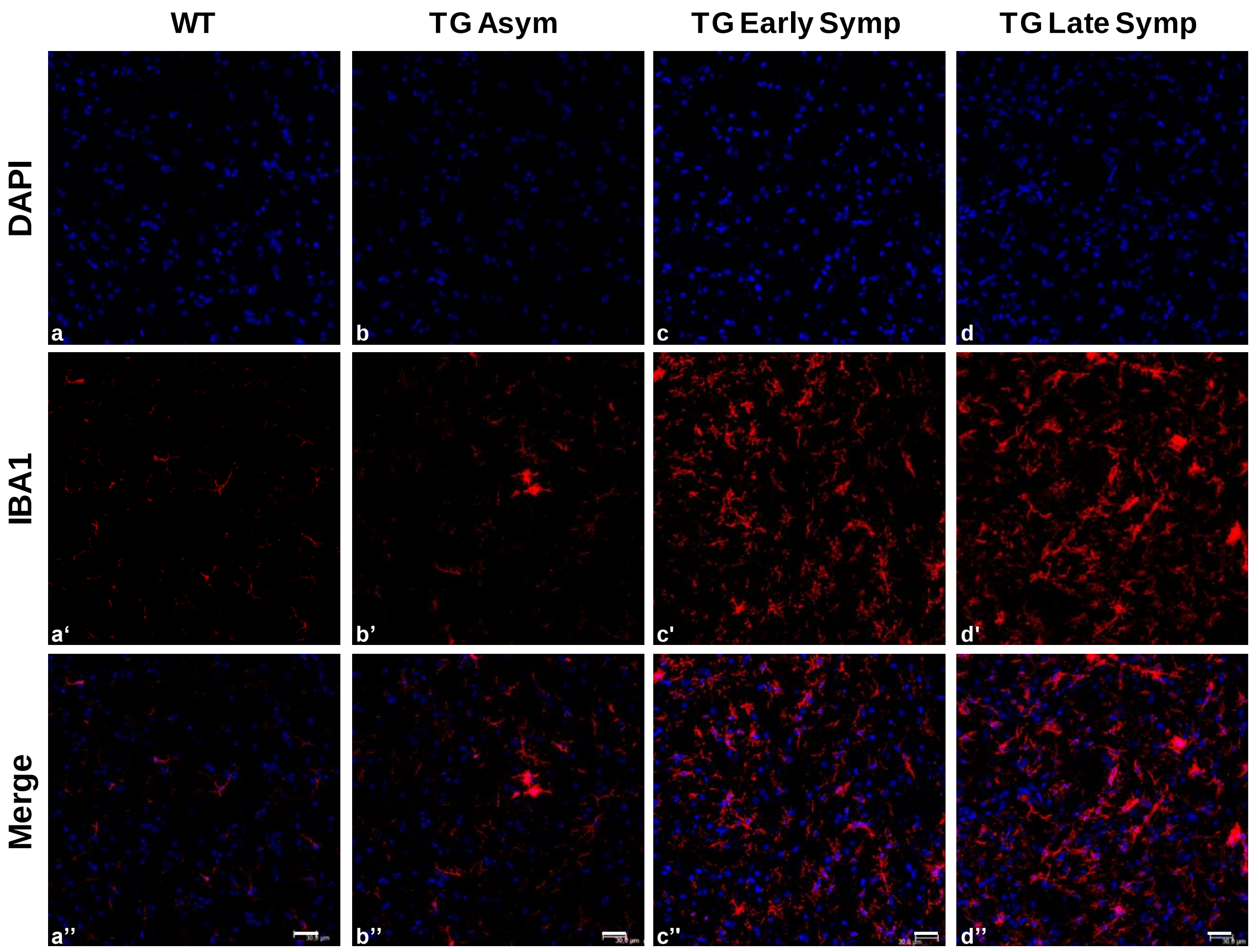

Figure S3. Microglial activation analysis.

Representative confocal images showing microglia activation in terms of IBA1-labeling (red) in the lumbar spinal cord of WT animals (a-a’’’), and Asym (b-b’’’), Early Symp (c-c’’’) and Late Symp (d-d’’’) TG mice. Nuclei are labelled with DAPI (blue). Scale bar= 30μm.

Disease Models & Mechanisms • Supplementary information
